# A Novel Vaccine Using Nanoparticle Platform to Present Immunogenic M2e against Avian Influenza Infection

**DOI:** 10.1155/2011/126794

**Published:** 2012-01-12

**Authors:** Sankhiros Babapoor, Tobias Neef, Christian Mittelholzer, Theodore Girshick, Antonio Garmendia, Hongwei Shang, Mazhar I. Khan, Peter Burkhard

**Affiliations:** ^1^Department of Pathobiology and Veterinary Science, University of Connecticut, 61 North Eagleville Road, Storrs, CT 06268, USA; ^2^Department of Molecular and Cell Biology, University of Connecticut, 91 North Eagleville Road, Storrs, CT 06269, USA; ^3^The Institute of Materials Science, University of Connecticut, 97 North Eagleville Road, Storrs, CT 06269, USA; ^4^M.E. Müller Institute, University of Basel, 50/70 Klingelbergstrasse, 4056 Basel, Switzerland; ^5^Institute for Medical Microbiology, University of Basel, 4003 Basel, Switzerland; ^6^^5^ Charles River SPAFAS, Inc., 106 RT 32 North Franklin, Storrs, CT 06254, USA; ^7^Department of Statistics, University of Connecticut, 215 Glenbrook Road, Storrs, CT 06269, USA

## Abstract

Using peptide nanoparticle technology, we have designed two novel vaccine constructs representing M2e in monomeric (Mono-M2e) and tetrameric (Tetra-M2e) forms. Groups of specific pathogen free (SPF) chickens were immunized intramuscularly with Mono-M2e or Tetra-M2e with and without an adjuvant. Two weeks after the second boost, chickens were challenged with 107.2 EID50 of H5N2 low pathogenicity avian influenza (LPAI) virus. M2e-specific antibody responses to each of the vaccine constructs were tested by ELISA. Vaccinated chickens exhibited increased M2e-specific IgG responses for each of the constructs as compared to a non-vaccinated group. However, the vaccine construct Tetra-M2e elicited a significantly higher antibody response when it was used with an adjuvant. On the other hand, virus neutralization assays indicated that immune protection is not by way of neutralizing antibodies. The level of protection was evaluated using quantitative real time PCR at 4, 6, and 8 days post-challenge with H5N2 LPAI by measuring virus shedding from trachea and cloaca. The Tetra-M2e with adjuvant offered statistically significant (*P* < 0.05) protection against subtype H5N2 LPAI by reduction of the AI virus shedding. The results suggest that the self-assembling polypeptide nanoparticle shows promise as a potential platform for a development of a vaccine against AI.

## 1. Introduction

Avian influenza (AI) is a devastating poultry disease with serious economic consequences to the commercial poultry industry. AI is also a significant public health concern because of recent highly pathogenic H5N1 avian influenza outbreaks causing also human deaths in Asia, Europe, and North Africa. According to the world health organization (WHO) update, 2011, since 2003, 520 confirmed cases of human infection with H5N1 have been reported, of which 307 died due to disease complications. However, other avian influenza viruses including low-pathogenic avian influenza (LPAI) can also be a risk to public health. For instance, the LPAI subtype H9N2 infection in chickens is mild to asymptomatic and easily overlooked. However, it shares similar receptor binding epitopes with human influenza viruses and can infect humans [1]. There is a risk for LPAI subtypes H5 and H7 to become high-pathogenic avian influenza (HPAI) viruses in chickens due to constant virus shedding and transmission to new birds within the flock or neighboring flocks [2, 3]. Vaccination is an effective way for prevention of viral diseases in poultry. However, routine vaccination against AI has not been widely practiced throughout the world mainly for surveillance reasons [1, 2]. When there is the desire for routine vaccination, constant reformulation of AI vaccines is required according to the circulating field virus, which can be cumbersome in the case of an immediate outbreak. Current vaccines against AI viruses can reduce mortality, clinical signs, shedding, and transmission of the virus in poultry, but they are not capable of preventing infection and virus replication [4].

The design of a universal influenza vaccine has been the major focus of researchers in the influenza vaccinology field. The external domain of matrix protein 2 (M2e) has been one of the main interests for the generation of a universal AI vaccine. The M2e is encoded by a separate open reading frame of segment 7 of the influenza virus genome, is located in the viral envelope, and projects from the surface of the virus as tetramers [5, 6]. The M2 is composed of 97 amino acids which forms 3 domains: the external domain, the transmembrane domain, and the internal domain. The external domain of M2 (M2e) is recognized by the host's immune system [7–9]. Initially, vaccination of ferrets with whole M-or M2-expressing recombinant vaccinia virus showed no evidence of protection [10]. However, later vaccine constructs using plasmid and recombinant salmonella expressing M or M2 induced significant protection in terms of reduction in virus growth and mortality in mice and chickens, respectively [11–13]. A multiple antigenic peptide construct containing M2e (M2e-MAP) induced strong M2e-specific antibody titers in the serum of mice and resulted in significant protection against influenza virus challenge [13]. Liang et al., 1994 [14] showed the importance of CD4^+^ T cells for nasal resistance and protection against the virus. It is assumed that M2e-specific memory T_h_ cells also may have an important role in protection against the virus in the nose and trachea of mice [13]. De Filette et al., 2005 [15] used the hepatitis B virus core particle (HBc) as a carrier and fused M2e (conserved region of human influenza A virus) to either the C-terminus of HBc or inserted it in the immune-dominant loop of HBc. Immunization of mice with this M2e-HBc vaccine was 100% protective against lethal challenge [15–17].

Antigenic epitopes of pathogens are peptides that are capable of inducing an immune response. However, their small size limits their immunogenicity. Therefore, usually a larger carrier protein, such as bovine serum albumin (BSA), keyhole limpet hemocyanin (KLH), or a virus-like particle (VLP), is required for optimal immunogenicity [18]. Structural organization of the epitope on the carrier is critical for inducing stronger immune responses. Denis et al., 2007 [19] demonstrated that a monomeric form of M2e peptide was not immunogenic and Huleatt et al., 2008 [20] tried to solve that problem by adding 4 copies of the M2e peptide in their platform. Here, we used peptide nanoparticles as a platform to display the M2e peptide to the host's immune system. These nanoparticles represent a novel type of repetitive antigen display system which allows presenting the M2e peptide in high density in both, either in its monomeric or its tetrameric form.

**Figure 1:**
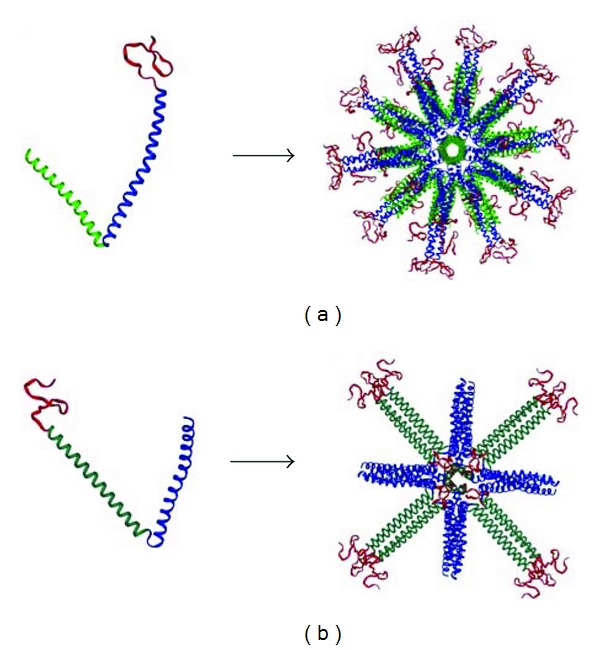
Computer model (a): the pentameric-trimeric architecture of Mono-M2e and the fully assembled icosahedral nanoparticle. (b) Tetra-M2e, with tetrameric-trimeric architecture, and the resulting octahedral nanoparticle. In both images, green: pentameric coiled coil, turquoise: tetrameric coiled coil, and blue: trimeric coiled coil. Red represents M2e in either its monomeric or tetrameric state.

 This idea was first presented in Raman et al., 2006 [21]; the monomeric peptide is composed of two coiled coils connected by a short linker region. The association between the coiled coils induces self-assembly of the monomers into spherical nanoparticles with either icosahedral or octahedral symmetry (Figure 1) according to our computer models. The potential for these nanoparticles to serve as platforms for vaccines is apparent. As opposed to live attenuated vaccines, there is no risk of infection within the vaccinated population [21]. Furthermore, the ease and speed of protein expression, purification, and self-assembly into nanoparticles reduce the cost and time of large-scale production. The concept has been successfully used for the design of malaria [22] and SARS [23] vaccines prototypes.

Here, we present the biophysical characterization of the nanoparticles and an immunological profiling using chickens as test animals. The results suggest that the self-assembling polypeptide nanoparticle shows promise as a potential vaccine against AI.

## 2. Materials and Methods

### 2.1. Nanoparticle Synthesis

The DNA coding for the nanoparticle constructs was prepared using standard molecular biology procedures. Shortly, plasmids containing the peptide monomers (Table 1) were constructed by cloning complementary oligonucleotides (CCCGGGGGGGCAGCGGCAGCCTGCTGACCGAAGTG- GAAACCCCGACCCGCAACGGCTGGGAATAATGAATTC) encoding the avian M2e epitope with flanking residues (ARGGSGSLLTEVETPTRNGWE**E) into the *Xma*I/*Eco*RI restriction sites of the basic SAPN expression construct to yield Mono-M2e. To generate Tetra-M2e, we first cloned the tetrameric oligomerization domain of tetrabrachion into the *Bam*HI/BssHII restriction sites of pPEP-T (Figure 5), before cloning complementary oligonucleotides (ATGCATCCCTGGTTCCGCGTGGAAGCCTGCTGACCG- AAGTGGAAACCCCGACCCGCAACGGCTGGGAATGCA- AATGCAGCGATAGCAGCGGATCC) coding for the slightly longer avian M2e sequence (HASLLTEVETPTRNGWECKCSDSSGS) including flanking residues into the N-terminal *Nsi*I/*Bam*HI restriction sites. The M2e-GCN4 construct was made by replacing the nanoparticle fragment of Tetra-M2e with the GCN4 sequence. The plasmids were transformed into *Escherichia coli* BL21 (DE3) cells, which were grown in Luria broth with ampicillin at 37°C. Expression was induced with isopropyl *β*-D-thiogalactopyranoside. Four hours after induction, cells were removed from 37°C and harvested by centrifugation at 4,000 ×g for 15 min. The cell pellet was stored at −20°C. The pellet was thawed on ice and suspended in a lysis buffer consisting of 9 M urea, 100 mM NaH_2_PO_4_, 10 mM Tris pH 8, 20 mM imidazole, and 0.2 mM Tris-2-carboxyethl phosphine (TCEP). Cells were lysed by sonication and the lysate was cleared by centrifuging at 30.500 ×g for 45 min. The cleared lysate was incubated with Ni-NTA Agarose Beads (Qiagen, Valencia, CA, USA) for at least 1 hour. The column was washed with lysis buffer and then a buffer containing 9 M urea, 500 mM NaH_2_PO_4_, 10 mM tris pH 8, 20 mM imidazole, and 0.2 mM TCEP. Protein was eluted with a pH gradient: 9 M urea, 100 mM NaH_2_PO_4_, 20 mM citrate, 20 mM imidazole, and 0.2 mM TCEP. Subsequent washes were done at pH 6.3, 5.9, and 4.3. Following the pH gradient, a gradient of lysis buffer with increasing imidazole strength was used to further elute the protein. Purity was assessed by sodium dodecyl sulfate polyacrylamide gel electrophoresis (SDS-PAGE) as shown in Figure 6.

 The protein solution was filtered with a 0.1 *μ*m polyvinylidene fluoride membrane filter (Millipore Billerica, MA, USA). Nanoparticle self-assembly was performed by dialysis into buffer containing 8 M urea, 20 mM Tris pH 7.5, 150 mM NaCl, and 5% glycerol, at a protein concentration of 0.1 mg/mL. This was followed by dialysis into the same buffer containing decreasing concentrations of urea: 6 M, 4 M, 2 M, 1 M, and two changes of the same buffer without urea. Following self-assembly, the nanoparticle solution was again filtered with a 0.1 *μ*m filter.

### 2.2. Dynamic Light Scattering

Dynamic light scattering experiments were carried out on a Zetasizer Nano S Instrument (Malvern, Worcestershire, UK), with a 633 nm He-Ne laser. All measurements were carried out at 25°C in a buffer containing 20 mM Tris pH 7.5, 150 mM NaCl, and 5% glycerol.

### 2.3. Transmission Electron Microscopy

Samples were negatively stained with 1% uranyl acetate (SPI Supplies, Westchester, PA, USA) and observed with a FEI Tecnai T12 S/TEM at an accelerating voltage of 80 kV (FEI, Hillsboro, Oregon). The peptide concentration of the constructs was about 0.05 mg/mL. 

### 2.4. Circular Dichroism

Samples were dialyzed into 20 mM sodium phosphate pH 7.5, 150 mM NaCl, and 5% glycerol and concentrated or diluted to a peptide concentration of about 0.13 mg/mL for Mono-M2e and about 0.05 mg/mL for Tetra-M2e. Circular dichroism measurements were performed at room temperature using an Applied Photophysics (Surrey, UK) Pi Star 180 spectropolarimeter, taking measurements from 200 to 250 nm. 

### 2.5. Viruses

The influenza virus used in the direct challenge AI study was A/Turkey/CA/D0208651-C/02 H5N2 low pathogenic. Influenza A/Turkey/Wisconsin/1/1966 H9N2 low pathogenic was used for hyperimmune serum production provided by Charles River Avian Vaccine Services (Storrs, CT). Viruses were grown and titered in 9-to 11-day-old embryonated specific pathogen-free (SPF) chicken eggs as previously described [24]. 

### 2.6. Animals and Experimental Groups

SPF P2a line (B19/B19) white Leghorn chickens eggs were obtained from Cornell University, Ithaca, NY. The eggs were hatched in the University of Connecticut Poultry Farm and after the hatch, the chickens were moved to the Office of Animal Research Services (OARS) facilities. After 2 weeks in the brooders with free access to water and a standard starter diet, the chickens were divided into groups, bled for baseline serology, transferred to isolators equipped with high-efficiency particulate air (HEPA) filters, and were provided commercial diets and water *ad libitum*.

### 2.7. Plaque Assay for Virus Neutralization

A previously described plaque reduction assay was modified and used to evaluate the virus neutralization activity of collected sera after vaccination [25]. Briefly, serum samples from each treatment group were pooled. An equal volume of a 1 : 10 dilution of pooled serum and LPAI subtype H5N2 was mixed and incubated for 30 min at 37°C. A commercially available anti-M2 antibody (ProSci-Inc, Poway, CA) was used in a 1 : 1000 dilution as a control for antibody activity. Chicken embryo kidney cell (CEKC) monolayers in 6-well plates were washed twice with prewarmed PBS and 400 *μ*L of the above mixture was added to the CEKC monolayer. The plates were incubated for 60 min at 37°C. Then, the inoculums were removed and after 2 washes with prewarmed PBS, they were overlaid with 0.8% agar (University of Connecticut Cell Culture Facility) in Minimum Essential Medium Eagle (MEM). After 72 h, the plates were checked for plaque formation and for further evaluation were fixed with 99% methanol and stained with crystal violet for plaque counting.

### 2.8. Measurement of M2e-Specific Antibody Response Using ELISA

The M2e epitopes, including the nanoparticle platforms with M2e epitopes (Tetra-M2e and Mono-M2e) and M2e linked to GCN4, (M2eN-GCN4), were used for coating of the ELISA plates. Briefly, individual wells of the flat-bottom 96-well Immulon 1B plates (NUNC/Thermo Fisher Scientific, Rochester, NY) were coated with 5 *μ*g/mL of tetrameric M2e (M2eN-GCN4) or the nanoparticle of interest. Antigen adhesion was allowed to proceed at 4°C overnight. Plates were rinsed with 2% Tween 20 in phosphate-buffered saline (PBS) (PBS/Tween 20 ThermoFisher) and blocked with a 3% BSA in PBS solution. Plates were incubated at 37°C for 3-4 h or 4°C overnight (preliminary studies showed that there was no difference in the result). After incubation, plates were rinsed 4 times with PBS/Tween 20 and incubated for 1 h at room temperature with the previously collected sera. Briefly, 2-fold serial dilutions of each serum sample were prepared in a PBS solution containing 0.2 to 0.5% BSA. Hyperimmune serum from previously infected birds with the LPAI subtype H9N2 or commercial anti-M2e antibody were used as positive controls; and sera from healthy, unvaccinated birds were used as a negative control. After appropriate washes, peroxidase-conjugated goat anti-chicken IgY (Sigma Aldrich,) was prepared in a 1 : 10,000 dilution in PBS and was added to each well and plates were incubated for an additional hour at room temperature. After subsequent rinsing, the plates were developed using 3,3′,5,5′ tetramethylbenzidine (TMB) peroxidase substrate (Thermo Fisher Scientific Inc., Rockford, IL) followed by a room temperature incubation period of 15 to 30 min. The absorbance was read in a SpectraMax 250 microplate reader (Molecular Devices, Sunnyvale, CA) at 450 nm.

### 2.9. Generation of pCR-M5 and In Vitro Transcription of M Gene

In order to generate a standard curve for real-time PCR, we transcribed standard RNA *in vitro* using T7 RiboMAX Express Large-Scale RNA Production System (Promega, Madison, WI). Briefly, RNA extraction was done by using Trizol reagent (Invitrogen, Carlsbad, CA) according to the manufacturer. The coding region of the M gene from the LPAI subtype H5N2 was amplified using previously described universal primers [26]. RT-PCR was performed using a Qiagen One-Step RT-PCR kit (Qiagen, Valencia, CA) according to the standard manufacturer's protocol. PCR products were visualized by electrophoresis through ethidium-bromide-stained (0.5 *μ*g/mL) 1.5% (40 mM Tris-Acetate pH 7.8, 0.1 mM EDTA) agarose gels under UV light.

 The amplified fragment was excised from the gel and cDNA was recovered from agarose gel using a QIAquick Gel Extraction Kit (Qiagen, Valencia, CA) according to the manufacturer's protocol and the purified DNA product was ligated with a pCR 2.1 vector (Invitrogen, Carlsland, CA) according to the manufacturer's protocol to generate the pCR-M5 plasmid. For further confirmation, PCR positive plasmids were sequenced in the DNA Biotechnology Facility of the University of Connecticut. Six micrograms of plasmid DNA was linearized using 6 units of the restriction enzyme *Bam H*I for 4 h at 37°C. Then, linearized DNA was used as a template in an *in vitro* transcription reaction with the T7 RiboMAX Express Large-Scale RNA Production System (Promega, Madison, WI) according to the manufacturer's recommendation. After the *in vitro* transcription reaction at 37°C for 1 h, the possible remaining plasmid DNA was digested by DNase I and purified RNA was quantified with a spectrophotometer* NanoDrop ND-1000 *(Thermo Fisher Scientific, Wilmington, DE). The copy numbers of purified RNA were determined using a previously described method [27] and was used for the generation of a real time standard curve.

### 2.10. Real-Time RT-PCR

In this study, real-time RT-PCR was performed using the previously published primers M+25: AGA TGA GTC TTC TAA CCG AGG TCG and M-124: TGC AAA AAC ATC TTC AAG TCT CTG for quantification of viral load [28]. RNA extraction was done on each swab sample followed by PCR in duplicate or triplicate using 5 *μ*L of RNA per each PCR reaction. Briefly, the *Power* SYBR Green RNA-to-CT *1-Step* Kit (Applied Biosystem, Foster City, CA) was used with a 20 *μ*L reaction mixture. For each PCR run, standards were designated for the plate and viral loads were calculated using fluorescence data acquired at the end of each annealing step. The amount of unknown sample was extrapolated based on the standard curve and was reported as viral copy number.

### 2.11. AI Challenge Study

Prior to vaccination and challenge study, a pilot study was performed to evaluate pathogenicity of the virus and determine the peak of virus shedding by measuring viral copy number in tracheal and cloacal sample of chickens at various time points postinfection with subtype H5N2 of low-pathogenicity avian influenza virus (LPAI). Briefly, thirty 2-week-old SPF chickens were divided into 3 groups of ten and were bled for baseline serology and transferred to isolators equipped with HEPA filters. At 8 weeks of age, chickens were inoculated both intranasally and intraocularly with 0.2–1 mL diluted allantoic fluid depending on the treatment group. Chickens in the low-dose challenge group received a 0.2 mL diluted allantoic fluid containing 10^6^ EID_50_, whereas the high-dose challenge group received 1 mL of allantoic fluid containing 10^7.7^ EID_50_. The third group of chickens remained in a separate isolator as a negative control. Tracheal and cloacal swabs were collected at 2, 4, 6, 8, 10, and 14 days postinfection using BD Universal Viral Transport (UVT) Kits (Becton, Dickinson, NJ) and Universal Viral Transport Polyester Swabs (Becton, Dickinson, NJ).

Upon determination of the peak of virus shedding and appropriate infectious dose in the pilot study, the vaccination and challenge trial was initiated. Briefly, 42 SPF chickens were divided into six groups of seven and received their first inoculation at 2 weeks of age followed by two boosters, two weeks apart, at 10 weeks and 12 weeks after hatch as described in Table 2. Preceding injection, nanoparticles were concentrated using Amicon Centrifugal Filter units with a 100 kDa MWCO (Millipore, Billerica, MA). Concentration was determined by absorbance at 280 nm and nanoparticle quality was assured by DLS. The nanoparticle vaccine constructs were emulsified with either Freund's complete adjuvant (prime) or Freund's incomplete adjuvant (boosters) and injected into the pectoral muscle of each chicken. Two weeks after the second booster, the birds, except for those in the negative control group, were challenged with 10^7.2^ EID_50_ LPAI subtype H5N2. Briefly, each bird received 1 mL allantoic fluid containing 10^7.2^ EID_50_ LPAI subtype H5N2 divided among the eyes, nasal cavity, and oropharynx, while temporarily blocking the fresh air delivery to the isolator. Fresh air was resumed after 5–10 min the following challenge of the last bird in the isolator. Although the clinical signs associated with LPAI viruses are rare, they were observed for possible clinical symptoms daily; and the presence of the symptoms and their severity was recorded.

Tracheal and cloacal swabs were taken from each bird at days 2, 4, 6, and 8 after challenge and they were placed in a 3.0 mL UVT tube (Becton, Dickinson, NJ). Blood samples from each bird were collected before each booster as well as two weeks after the second booster prior to challenge. Each blood sample was collected in a separating blood tube and serum was separated by placing the tubes at 37°C for 1 h then at room temperature overnight followed by a 5 to 10 min centrifugation at 1000 rpm at 4°C. Then, the collected serum samples were stored at −20°C until analysis.

## 3. Results

### 3.1. Nanoparticle Design

An obvious model for a self-assembling protein particle is a viral capsid. The capsids of spherical viruses often have icosahedral symmetry, due to their need to build a large encapsulating structure from many copies of the same, or only few, capsid proteins. An icosahedron is the most efficient way to accomplish this. By utilizing pentameric and trimeric coiled coils, we have built a self-assembling nanoparticle which uses the threefold and fivefold symmetry of an icosahedrons [21]. The pentameric coiledcoil motif of the monomer is taken from Cartilage Oligomeric Matrix Protein (COMP) and the trimer is a *de novo* designed coiled coil. Self-assembly occurs when the coiled-coil domains of different monomers associate, forming the icosahedral nanoparticle (Figure 1). A nanoparticle with this sort of architecture can then be used as a vaccine platform by extending the ends of the monomer with an epitope sequence. The Mono-M2e species of nanoparticle follows this plan (Table 1). As a result, it repetitively displays a monomeric form of M2e on the surface of the nanoparticles. The M2e peptide on the icosahedral nanoparticles lacks its C-terminal five residues to avoid problems with disulfide crosslinking that presumably require the native tetrameric conformation for proper formation. Although the simplest icosahedral particle with T1 icosahedral symmetry is made from 60 polypeptide chains, it may also be possible that Mono-M2e particles possess higher triangulation numbers, resulting in particles with an even greater molecular mass.

 On the other hand, the native conformation of M2 is a tetramer. Hence, to elicit conformationally specific antibodies, the M2e antigen displayed by a vaccine particle should ideally be tetrameric. With that in mind, we designed the Tetra-M2e peptide (Table 1). Instead of a pentameric coiled coil, this polypeptide uses the tetrameric coiledcoil motif from the protein tetrabrachion [29]. Self-assembly using this peptide would result in a nanoparticle with threefold and fourfold symmetry axes or octahedral symmetry. As opposed to the larger icosahedral Mono-M2e, this octahedral particle would only have 24 polypeptide chains. In addition, the epitope is now constrained to its native tetrameric conformation. The full-length M2e contains two cysteine residues. The formation of disulfide bridges between the cysteines of adjacent chains under oxidizing conditions is thought to stabilize their tetrameric conformation.

 The speed and ease of protein expression and purification, as well as of the self-assembly process, contribute to the overall viability of this technology as a vaccine platform. To facilitate purification, we have included polyhistidine tags at the N-terminal ends of the peptides.

To enable detection of antibodies against tetrameric M2e, the peptide M2eN-GCN4 was designed (Table 1). M2e is linked to a GCN4, a coiled coil whose oligomerization state can be determined by the identity of amino acid residues in key a and d positions of the coiled coil. In this case, the tetrameric version of GCN4 was used [30]. By affixing M2e to a tetrameric protein, we can constrain it in its tetrameric conformation. The effect is similar to that experienced by the ends of the tetrameric coiled coil from tetrabrachion of the Tetra-M2e nanoparticle. However, the coiled coil sequence is different. This guarantees that any antibodies bound to M2e-GCN4 are specific for the tetrameric version of M2e and not against the coiled coil or other parts of the nanoparticle.

### 3.2. Size Distribution

**Figure 2:**
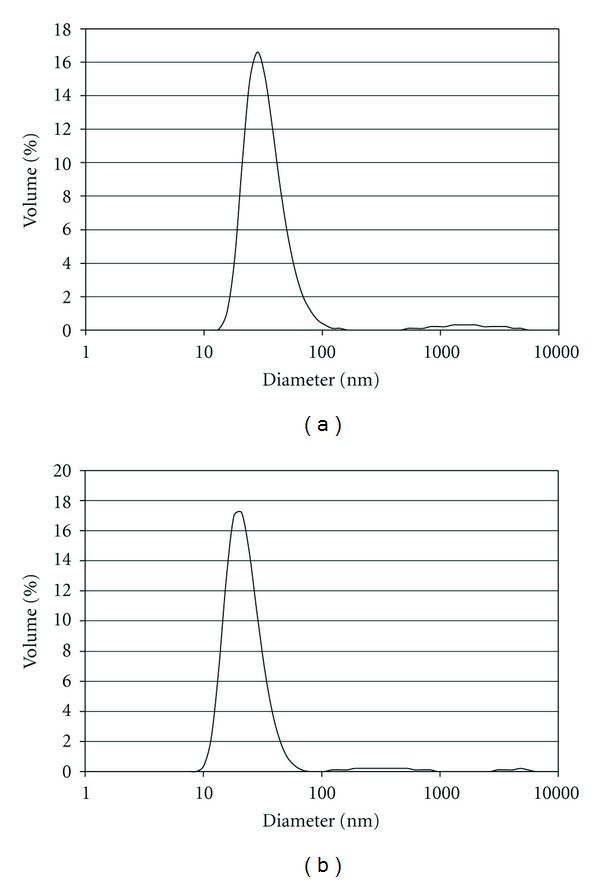
Dynamic light scattering. Size distributions of (a) Mono-M2e (Peak = 34.5 nm) and (b) Tetra-M2e (Peak = 22.9 nm) as measured by dynamic light scattering. The experiments were performed in the buffer 20 mM Tris pH 7.5, 150 mM NaCl, and 5% glycerol.

Dynamic light scattering revealed that Mono-M2e formed particles whose hydrodynamic diameters have a distribution which peaks at 34.5 nm, while the distribution of Tetra-M2e peaks at 22.9 nm (Figure 2). It is also noteworthy that the size distribution peak of Mono-M2e is broader than that of Tetra-M2e, suggesting that the former has a higher degree of polydispersity.

**Figure 3:**
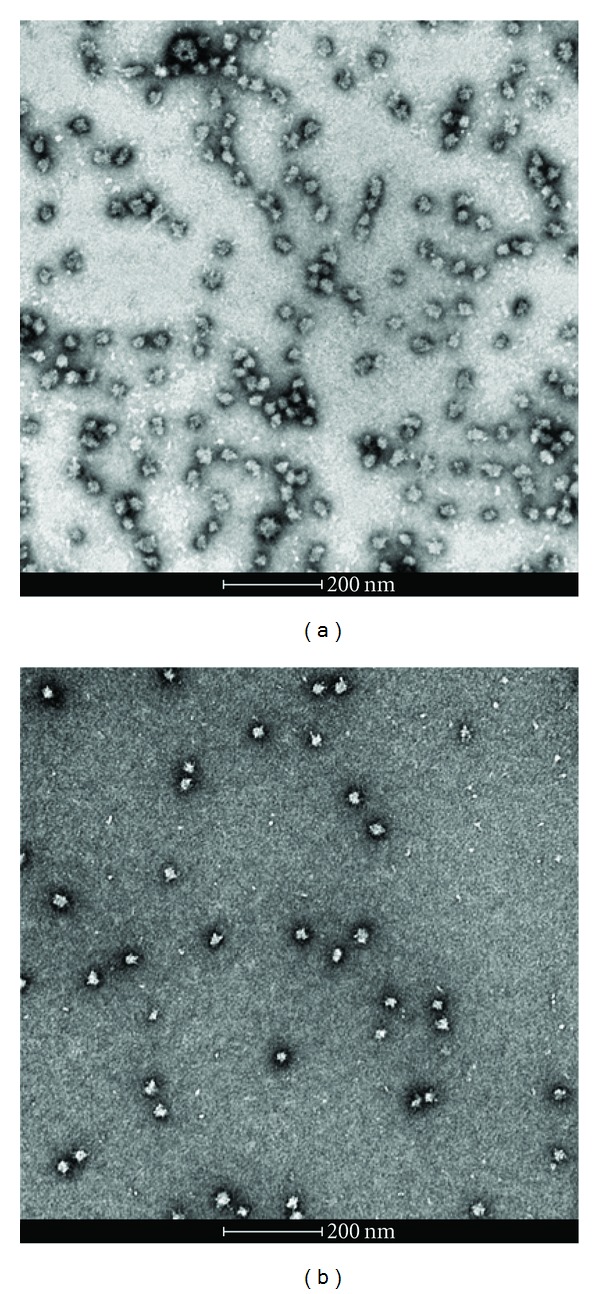
Transmission electron microscopy: (a) Mono-M2e (peptide concentration = 0.05 mg/mL) and (b) Tetra-M2e (peptide concentration = 0.04 mg/mL). Samples were negatively stained with 1% uranyl acetate. The samples were in the buffer 20 mM Tris pH 7.5, 150 mM NaCl, and 5% glycerol.

The results were confirmed by transmission electron microscopy (Figure 3). We can see that nanoparticles were formed and that their diameters are comparable with those measured by dynamic light scattering. It can be seen from the micrographs that neither Mono-M2e nor Tetra-M2e form nanoparticles with perfectly spherical morphology. This may in some way explain the polydispersity observed by dynamic light scattering.

### 3.3. Secondary Structure

**Figure 4:**
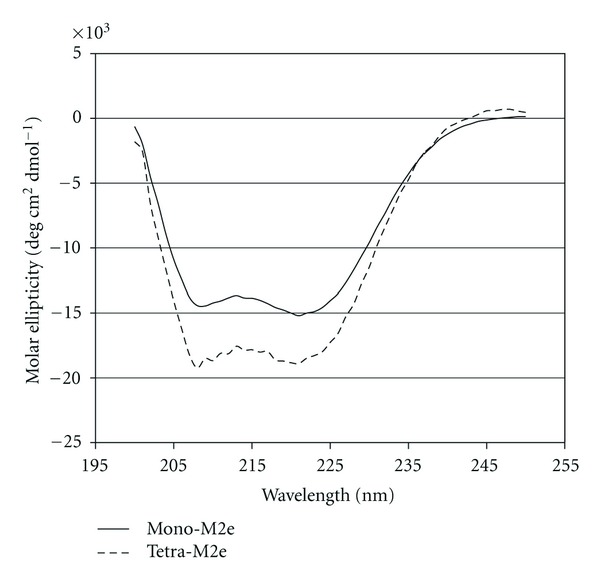
Circular dichroism. (a) Mono-M2e at a concentration of 0.132 mg/mL. (b) Tetra-M2e at a concentration of 0.048 mg/mL. Experiments were performed in 20 mM sodium phosphate pH 7.5, 150 mM NaCl, and 5% glycerol.

**Figure 5:**
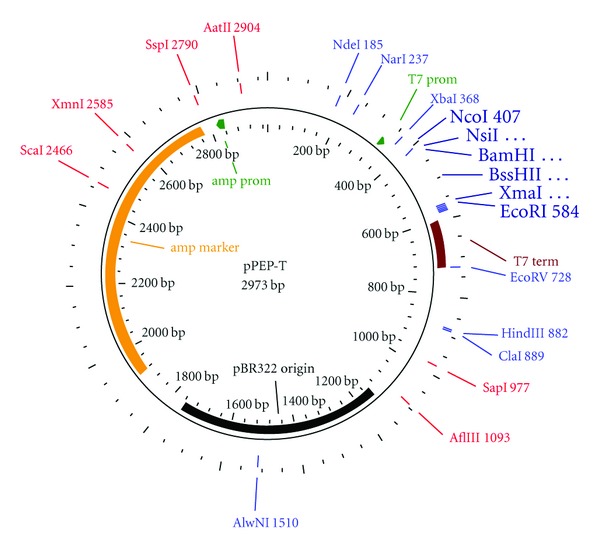
Vector map of pPEP-T. The insertion sites used for subcloning are shown in larger letters. For the external insertion sites, NcoI and EcoRI, the nucleotide numbers of the original vector are indicated, while for the internal restrictions sites, NheI, BamHI, and XmaI, the nucleotide numbers varied, depending on the construct.

**Figure 6:**
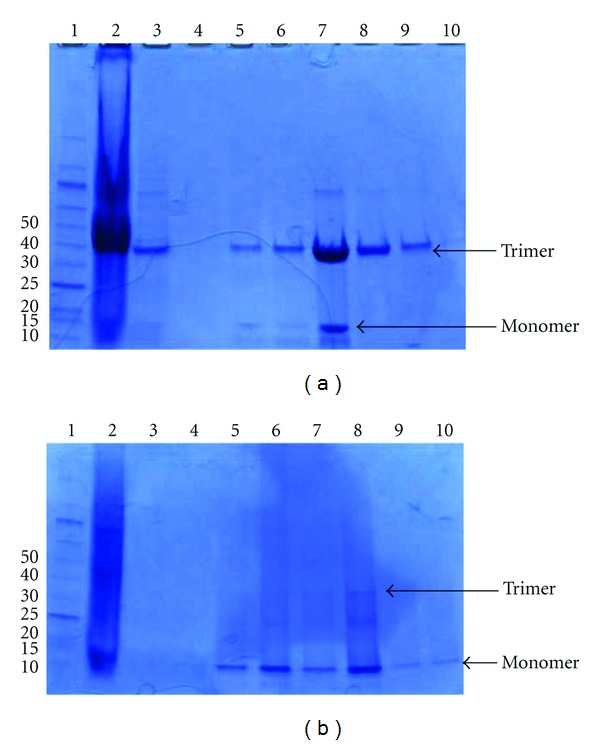
SDS-PAGE of SAPN Constructs. (a) Purification of Mono-M2e. (1) Molecular weight marker protein ladder (New England Biolabs, Ipswich, MA). (2) Flow through. (3) wash with lysis buffer. (4) Wash with high-phosphate buffer. (5) pH 6.3 wash. (6) pH 5.9 wash. (7) pH 4.3 wash. (8) Wash with 250 mM imidazole buffer. (9) Wash with 500 mM imidazole buffer. (10) Wash with 1000 mM imidazole buffer. (b) Purification of Tetra-M2e. (1) Molecular weight marker protein ladder. (2) Flow through. (3) pH 6.3 wash. (4) pH 5.9 wash. (5) pH 4.3 wash. (6) Wash with 100 mM imidazole buffer. (7) Wash with 250 mM imidazole buffer. (8) Wash with 500 mM imidazole buffer. (9) Wash with 1000 mM imidazole buffer fraction # 1. (10) Wash with 1000 mM imidazole buffer fraction no. 15.

The double minima found by circular dichroism confirm the alpha helical structure of the nanoparticles (Figure 4). It appears that Tetra-M2e exhibits this behavior much less than Mono-M2e. This may be partly due to the larger M2e epitope sequence in the Tetra-M2e peptide as compared to that used in Mono-M2e.

### 3.4. Testing Neutralization Capability of Anti-M2e Antibody

The plaque reduction assay performed by using pooled serum from chickens inoculated with Tetra-M2e did not show a significant (*P* > 0.05) difference compared to control nonvaccinated chicken serum and commercial anti-M2e antibody.

### 3.5. AI Challenge Study

**Figure 7:**
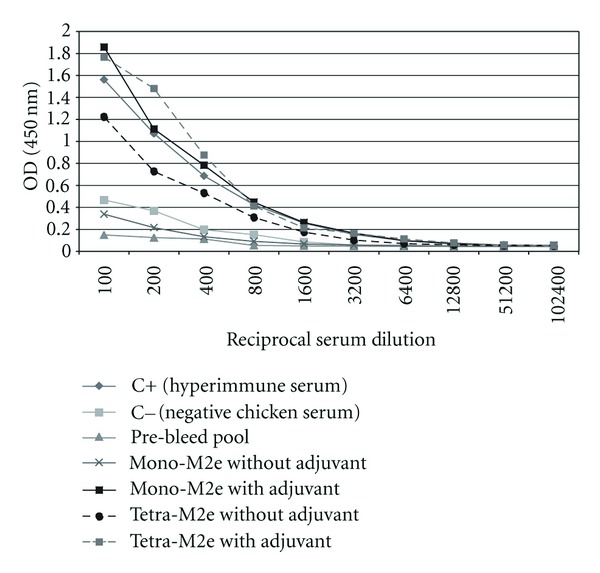
Serum Anti-M2e antibody level in chicken serum after 3rd inoculation. Antibody response to immunization was measured using M2e-GCN4 (5 mg/mL) coated ELISA plates and OD was reported at 450 nm.

**Figure 8:**
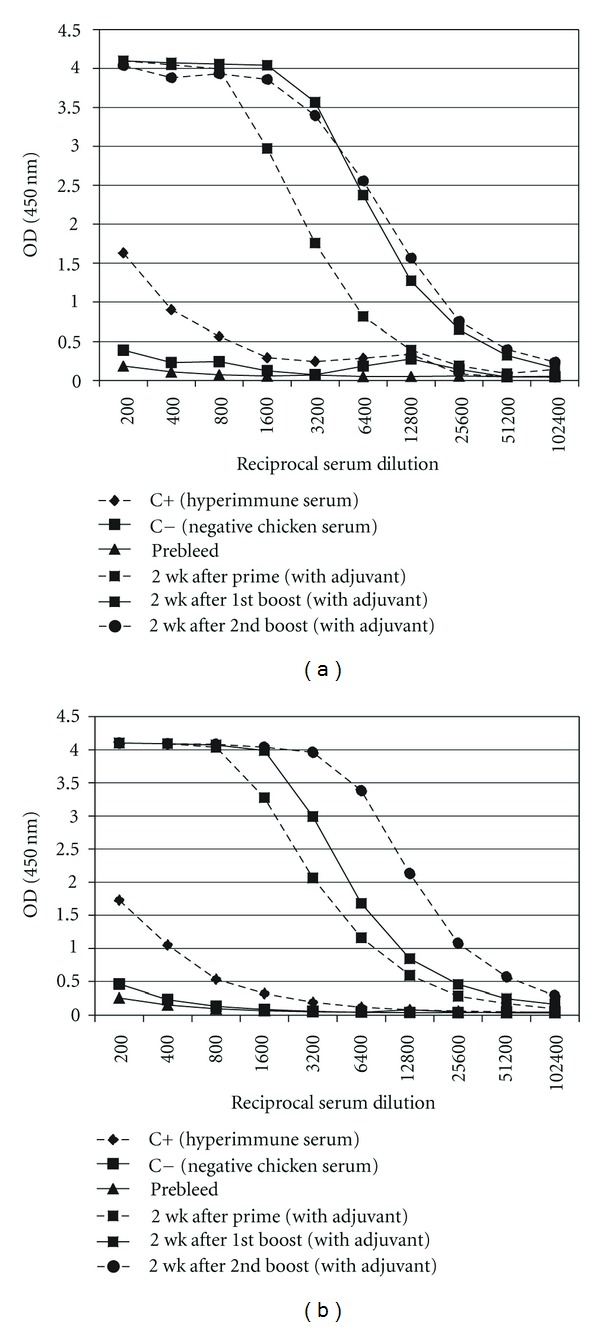
Serum Anti-M2e antibody level in chicken serum after each inoculation. Antibody response to immunization was measured using Mono-M2e (5 *μ*g/mL) (a) and Tetra-M2e (b) coated ELISA plates to observe the antibody titer after each boost. Prebleed, prime, first boost and second boost of groups vaccinated with Mono-M2e and Tetra-M2e with adjuvant are shown in the graph.

**Figure 9:**
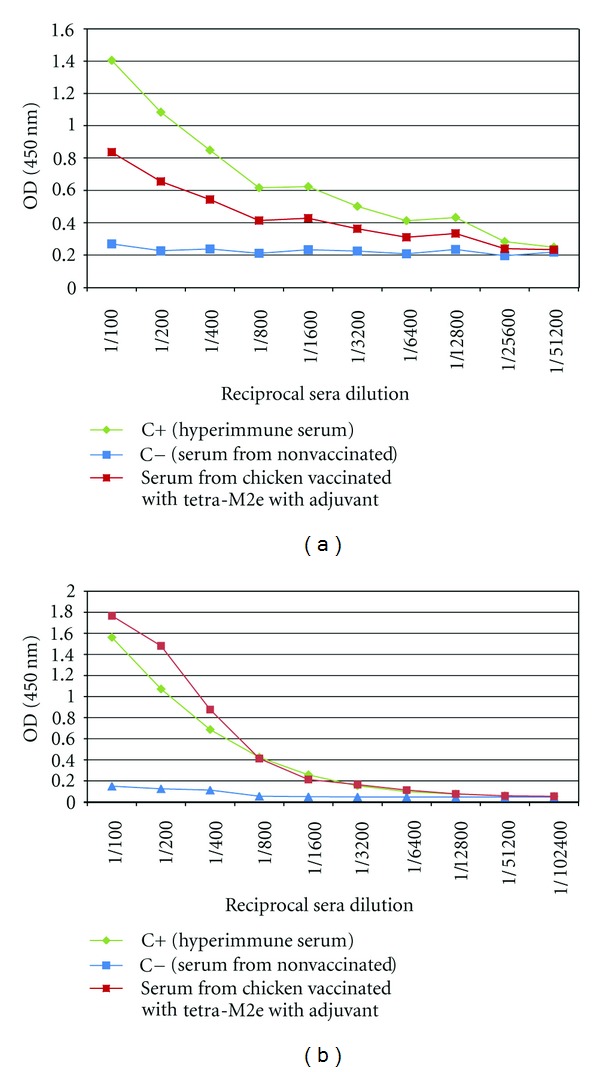
Evaluation of virus binding capability of the postvaccination serum antibody. Serum from chicken vaccinated with tetra-M2e was evaluated after 2nd boost over night at 4°C. Control positive is a serum collected from infected chickens with H9N2 (Charles River SPAFAS, Inc.). (a) Plate coated with heat inactivated virus. (b) Plate coated with GCN-M2e.

**Figure 10:**
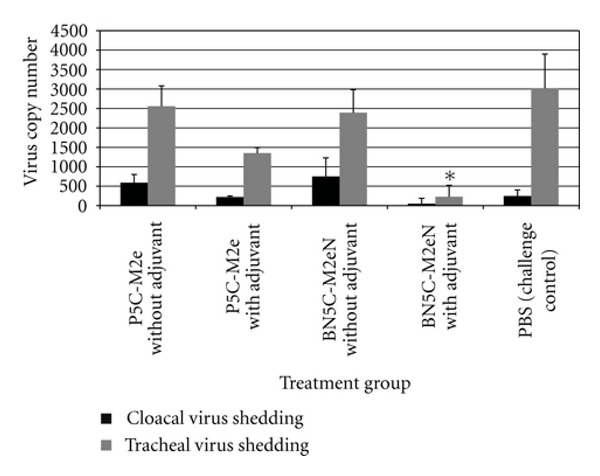
Virus shedding at day 8 after challenge (postinfection). Cloacal virus shedding and tracheal virus load were measured for Mono-M2e (monomeric M2e), TetraM2e (tetrameric M2e), PBS, and control negative. *Black*: cloacal shedding *gray*: tracheal shedding. ∗was significantly different (*P* < 0.05).

**Figure 11:**
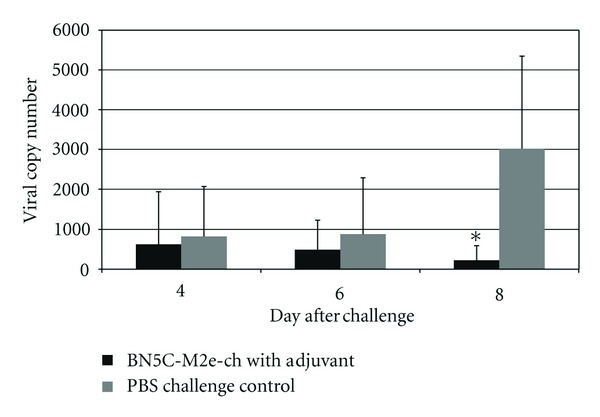
Virus shedding at 4, 6, and 8 days post challenge (post infection). Tracheal virus load was measured for Tetra-M2e (tetrameric M2e) with adjuvant (*black*) in comparison with PBS challenge control (*gray*) at 4, 6, and 8 days post challenge. ∗was significantly different (*P* < 0.05).

The anti-M2e immune response was monitored by determining the titer of the M2e-specific IgY at three different time points (2 weeks after each inoculation). Chickens after each inoculation developed high levels of antibody against the injected construct and anamnestic response clearly was seen when the plates were coated with Mono-M2e and Tetra-M2e nanoparticles and M2e-GCN4 (tetrameric M2e), respectively (Table 1, Figures 7 and 8). For further investigation of the antibodies, the level of M2e specific antibody was measured using plates coated with tetrameric M2e-GNC4 peptide to evaluate the specific antibody against tetrameric M2e rather than the whole particle. The result of this study indicated that in chickens, after the second booster, the antibody levels are not at the same level as our previous results in mice with the same backbone but a different (malaria) epitope had been shown [23]. The dose level was also higher than what was shown to be required in mice. This could be because of the lower haplotype-specific immunogenicity of the particles in chickens, the route of administration in mice (intraperitoneal and intranasally), the body weight of the mice compared with chickens, and different immune system repertoires of mammalian and avian species. In future studies, changing the administration route can be another approach to reducing the dose of vaccine construct. We also coated the plate with inactivated purified virus to observe seroconversion of the chickens after challenge with the virus at 2 weeks after the last boost. Results indicated that whole virus response was higher as expected with hyperimmune serum (Figure 9(a)), however, ELISA response from chicken vaccinated with tetra-M2e and with whole virus reacted similarly on GCN-M2e coated plate (Figure 9(b)). The protective efficacy of the anti-M2e antibody responses induced by different constructs was assessed by evaluation of viral shedding post challenge. To determine the peak of shedding, viral copy number was measured in tracheal and cloacal sample of chickens after infecting them with a LPAI virus A/Turkey/CA/D0208651-C/02 H5N2. Briefly, in a pilot study chickens were infected with 10^6^ EID_50_ and 10^7.7^ EID_50_ of the virus and by using real time RT-PCR, tracheal and cloacal virus shedding was evaluated at 2, 4, 6, 8, 10, and 14 days postinfection. The 10^7.7^ EID_50_ was found to be a good indicator of virus shedding. First, a significant (*P* < 0.05) rise in tracheal and cloacal virus shedding was observed at days 4 and 8 postinfection with a magnified peak of tracheal shedding at 8 days postinfection. Taking into account these results, in the vaccination and challenge trial, individual tracheal and cloacal swabs were collected at 4, 6, and 8 days after challenge for better determination of protection. The results of the real-time RT-PCR testing of cloacal and tracheal swab samples taken on day 8 after vaccination and challenge study are shown in Figure 10. We determined viral loads in tracheal and cloacal swabs samples on day 8 following challenge with 10^7.2^ EID_50_ LPAI subtype H5N2. Reduction of cloacal and oropharyngeal shedding in vaccinated birds was significant in chickens vaccinated with Tetra-M2e with Freund's adjuvant. Virus shedding was evaluated at day 4 and day 6 after challenge; the swabs were tested for virus load (Figure 11). It is seen that virus shedding reduction starts at day 4 post infection with a significant decrease at day 8 post infection.

## 4. Discussion

Currently available vaccines induce antibodies against specific field strains or closely related avian influenza strains. Most of these vaccines are killed virus vaccines that induce short-lived immunity and are lacking a broad cross-reactive humoral immune response. Recently, the generation of a universal influenza vaccine using conserved peptide regions among several influenza virus strains has been an area of interest in the human influenza vaccine field. M2e is a highly conserved region among influenza viruses and it has been studied as a possible universal vaccine candidate against human influenza virus infection [16, 17].

In the present study, protection efficiency of two different nanoparticle constructs harboring M2e was studied as possible vaccine candidates for low-pathogenicity avian influenza infection. Biophysical analysis confirms that they are of relatively regular shape and size, but there is some degree of heterogeneity. Though molecular weight measurements still remain to be carried out and we have no high resolution structural data, we assumed that nanoparticles assembled in a state close to what was expected, that is, icosahedral and octahedral nanoparticles, respectively. This will repetitively display M2e in both, either in its monomeric or its tetrameric form.

There is speculation as to how the polyhistidine tag at the N-terminal end of the monomers may affect the self-assembly process, the final nanoparticle structure, or the immunogenicity of the vaccine, but attempts at producing his-tag free versions of the nanoparticle constructs either did not reliably provide pure protein or never adequately self-assembled. Similarly, we attempted to include CD4 T cell epitopes to increase the immune response, but this also interfered with nanoparticle formation.

Since many variables can affect the host's virus shedding and the course of disease [31, 32], prior to evaluation of vaccine constructs, the pathogenicity after challenge with the LPAI subtype H5N2 virus was evaluated. A biphasic virus shedding was observed in this study. For the LPAI subtype H5N2, the peaks for tracheal and cloacal shedding were at days 4 and 8 postinfection. 

The Tetra-M2e vaccine construct provided a significant viral load reduction at the peak of viral shedding in immunized chickens. Chickens immunized with Tetra-M2e that harbors the tetrameric M2e with Freund's adjuvant showed a clear reduction in cloacal and tracheal excretion of LPAI compared to challenge control groups. The results of immunization with Mono-M2e with adjuvant and Tetra-M2e without adjuvant were also promising and by improving both B- and T-cell epitopes of those constructs, desirable results may be obtained. In vaccine design, repetitive B-cell epitope display is considered a strategy for improving the humoral immune response [33, 34]. In addition to repetitive antigen display on the nanoparticle, we were able to present M2e in its native tetrameric conformation. The correlation between high protection and antibody response specific for tetrameric M2e elicited by Tetra-M2e supports our assumption of tetrameric M2e presentation. The results of our studies show that tetrameric M2e stimulates a more specific immune response compared to the monomeric presentation and induces a significant protection against homologous virus challenge. The fact that a large portion of the antibody response is directed against the carrier and not only against the epitope(s) (Figures 7 and 8) can be explained by the fact that significant portions of the core of the nanoparticles are also exposed to the immune system (compare Figure 1) and hence, these portions will also induce a significant immune response.

 In this study, we showed that anti-M2e antibodies are not neutralizing antibodies; however they are capable of binding to the M2 proteins that are abundantly presented on the surface of the infected cells (data not shown). These can describe an efficient delayed clearance of the virus in M2e vaccinated chickens based on the previously described NK cell involvement in ADCC [35]. Significant improvement of virus clearance in vaccinated chickens with tetrameric M2e may be considered in a new vaccination strategy by vaccinating chickens with both a killed vaccine and a nanoparticle vaccine in order to provide robust protection, cross-reactive immunity, and clearance in case of emerging new strains of the virus.

However, there remains the risk that such vaccination may cause a long-term persistence of HPAI in poultry flocks, because the vaccine could not prevent the viral infection but rather suppresses the symptoms of HPAI virus-infected chickens by reducing the virus shedding in chicken. Thereby, especially in the case of HPAI infection, the vaccination may make the infection less visible and the eradication of virus more difficult, and consequently it may provide a good opportunity for HPAI virus to survive and persist in poultry flocks for a long time. For this reason, we plan to design new nanoparticle constructs that also contain fragments of hemagglutinin in addition to the M2e domain. Immunization would then result in the generation of neutralizing hemagglutinin-specific antibodies in addition to the disease modulating M2e-specific antibodies.

In this study, we evaluated a new approach to immunizing chickens against AI that uses a nanoparticle platform to carry an antigenic epitope. Further designing and testing of new nanoparticle vaccines should demonstrate that they are effective tools for stimulation of an immune response against M2e and other B- or T-cell epitopes. Therefore, application of the nanoparticle platform facilitates the development of a new generation of vaccines that harbor conserved epitopes of avian influenza viruses and would not be rendered ineffective by viral mutations such as antigenic shifts and drifts. The nanotechnology described here offers the opportunity to rapidly produce new vaccines according to the emergence of new strains of influenza virus without going through the time-consuming steps of production currently used in manufacturing commercial influenza vaccines. For future studies, the chicken's LPAI infection model needs to be improved to evaluate clinical signs and higher virus shedding. This may help to better evaluate virus shedding, specifically cloacal virus shedding. Also, vaccination and HPAI challenge may be used to evaluate the vaccine efficiency in protection against high-pathogenicity AI viruses.

## Figures and Tables

**Table 1:** Summary of self-assembling nanoparticle peptide sequences.

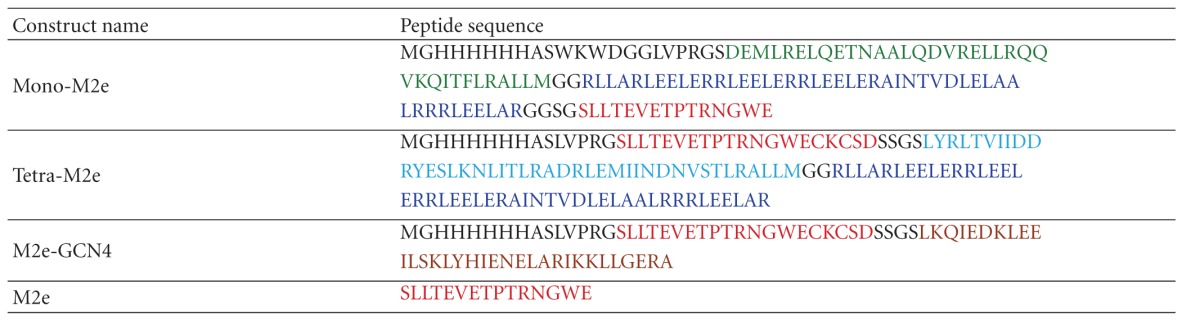

The peptide Mono-M2e is composed of a pentameric coiled coil (green) and a trimeric coiled coil (blue). Tetra-M2e uses the same trimer but has a tetrameric coiled coil (turquoise). In both sequences, the M2e epitope is shown in red. Other amino acid residues, such as linkers and his-tags, are shown in black. M2eN-GCN4 consists of M2e attached to the tetrameric GCN4 coiled coil, shown in brown. Monomeric M2e, used for ELISA, coating is shown in red.

**Table 2:** Overview of immunization regimen.

Group	Dose (*μ*g)	Inoculum	Adjuvant	Vaccination	Challenge
G1	75	Mono-M2e	−	+	+
G2	75	Mono-M2e	+	+	+
G3	75	Tetra-M2e	−	+	+
G4	75	Tetra-M2e	+	+	+
G5	—	PBS	−	−	+
G6	—	Nonvaccinated	−	−	−

Complete Freund's adjuvant was used for the priming vaccine followed by incomplete Freund's adjuvant for boosters. Challenge with LPAI subtype H5N2 was performed at 8 weeks of age using oculonasal route.

## Abbreviations

AI: Avian influenza
M2e: Ectodomain of matrix protein 2
LPAI: Low pathogenicity avian influenza
HPAI: High pathogenicity avian influenza
SPF: Specific pathogen free.
